# Effects of dietary supplementation of herbal active ingredients promoting insulin-like growth factor-1 secretion on production performance, egg quality, blood hematology, and excreta gas emission in laying hens

**DOI:** 10.5713/ab.20.0762

**Published:** 2021-06-23

**Authors:** De Xin Dang, Yi Hyung Chung, In Ho Kim

**Affiliations:** 1 Department of Animal Resource and Science, Dankook University, Cheonan 31116, Korea; 2 Jeonbuk Institute for Food-Bioindustry, Jeonju, 54810, Korea

**Keywords:** Egg Quality, Herbal Extract, Insulin-like Growth Factor-1, Laying Hen, YGF251

## Abstract

**Objective:**

The purpose of this study was to evaluate the effects of supplementing herbal active ingredients (YGF251) which can promote the secretion of insulin-like growth factor-1 (IGF-1) in the diet on production performance, egg quality, blood hematology, and excreta gas emission in laying hens.

**Methods:**

A total of 288 ISA Brown (41-week-old) laying hens with an initial body weight of 1.83±0.68 kg were randomly assigned to 1 of 4 dietary treatments in a randomized block design based on body weight. Each treatment had 12 replicate cages having 6 adjacent cages per replicate (hens are kept in cages alone). The experimental period was 35 days. Dietary treatments were based on the corn-soybean meal-wheat-based basal diet and supplemented with 0.00%, 0.05%, 0.10%, or 0.15% YGF251.

**Results:**

There was a linear increased egg weight in weeks 1 to 5 (p<0.05), egg mass in week 1 (p<0.05) and weeks 1 to 5 (p<0.05), egg strength on day 7 (p<0.05), 21 (p<0.01), and 35 (p<0.01), eggshell thickness on day 21 (p<0.05) and 35 (p<0.01), haugh unit on day 21 (p< 0.01) and 35 (p<0.05), serum IGF-1 concentration on day 21 (p<0.05) and 35 (p<0.01), and serum total protein concentration on day 35 (p<0.05) were observed with the supplementing YGF251 increased in the diet, while feed conversion ratio in weeks 1 to 5 (p<0.05) and excreta ammonia emission (p<0.01) decreased linearly with the dose of YGF251 increased.

**Conclusion:**

Dietary supplementation of YGF251 positively affected the production performance and egg quality of laying hens through increasing serum IGF-1 concentration in a dose-dependent manner. Moreover, YGF251 supplementation improved barn environment by reducing excreta noxious gas emission.

## INTRODUCTION

Eggs create considerable value for animal husbandry as animal products. Antibiotics as performance enhancers in layer production have primarily been applied to improve utilization of feed and reduce pathogenic bacteria in the gut, thereby improving production performance [1]. However, due to the increase of multiple resistance bacteria and the decrease of consumers acceptance, the use of antibiotics has been gradually prohibited [2]. In recent years, phytogenic feed additives have attracted more and more attention because of their safety and environmental friendliness [2]. Some researchers have reported the positive effects of herbal extract supplementation on production performance and egg quality of laying hens [3,4].

In this study, the herbal active ingredient (YGF251), which promotes the secretion of insulin-like growth factor-1 (IGF-1), was extracted from the herbal mixture of Shady Jerusalemsage (*Phlomis umbrosa Turcz*), Wilford Swallowwort (*Cynanchum wilfordii Hemsley*), Ginger (*Zingiber officinale Rosc*), and Balloonflower root (*Platycodi Radix*). The ability of YGF251 to promote the secretion of IGF-1 has been proved by Choi et al [5] and Kim et al [6]. Recently, it has been reported that dietary supplementation of YGF251 had positive effects on the growth performance and reproductive performance in animals [7,8]. When used in poultry husbandry, Begum et al [9] reported that dietary supplementation of 0.05%, 0.10%, or 0.15% YGF251 increased body weight, energy retention, femur length, and serum IGF-1 concentration of broiler chicks. Moreover, Cos et al [10] noted that the herbal active ingredients which promote IGF-1 secretion have the characteristics of phytoestrogens. When supplemented in the diet, they can have a series of beneficial effects on the reproductive performance of birds. It has been reported that dietary supplementation of the above herbal-derived active ingredients could promote the secretion of IGF-1 *in vivo*, thus improving production performance and egg quality in ducks [11] and quails [12].

Therefore, we hypothesized that supplementing YGF251 to the diet of laying hens would have positive effects on the production performance and egg quality because it has the properties for promoting the secretion of IGF-1 *in vivo*. However, since there are no studies to evaluate the effects of dietary supplementation of YGF251 on the performance of laying hens, it is necessary to evaluate its effects on production performance, egg quality, blood hematology, and excreta gas emission in laying hens.

## MATERIALS AND METHODS

The experiment was conducted at the poultry experimental unit of Dankook University (Anseodong, Cheonan, Choongnam, Korea). The study protocol (No. DK-2-1826) was approved by the Animal Care and Use Committee of Dankook University.

### Source of herbal extract

The plant extract used in the present study was a commercial product obtained from Doosan Feed Inc. (Bucheon, South Korea), which was extracted from the herbal mixture including Shady Jerusalemsage (*Phlomis umbrosa Turcz*), Wilford Swallowwort (*Cynanchum wilfordii Hemsley*), Ginger (*Zingiber officinale Rosc*), and Balloonflower root (*Platycodi Radix*) [8].

### Experiment design, animals, and housing

A total of 288 ISA brown laying hens (41-week-old) with an initial body weight of 1.83±0.68 kg were used for a five-week trial. All birds were caged individually and randomly assigned to 4 treatments based on body weight. There were 12 replicate cages per treatment and 6 hens per replicate. The diets of layers were in mash form and formulated by meeting the nutritional requirements recommendations of the NRC [13]. The dietary treatment consisted of a basal diet (Table 1), and a basal diet supplemented with 0.05%, 0.10%, or 0.15% YGF251.

Hens were housed in natural ventilation and program mable lighting equipped room and individually reared in a 38×50×40 cm adjacent steel cage, which was installed with nipple drinkers, common trough feed, and egg collection plate. Throughout the whole experimental period, the average ambient temperature was 23°C. Moreover, the daily photo-period was subjected to 16 hours (05:00 to 21:00) light (the light intensity was 5.2 lx) and 8 hours dark. The feed and water were provided *ad libitum* to hens throughout all periods of the experiment.

### Sampling and measurements

The number and weight of eggs and feed intake were recorded daily on a replication basis to calculate the average daily feed intake and egg production rate. Egg mass was calculated as egg weight × egg production. The feed conversion ratio (FCR) was calculated as grams of feed intake per gram of egg mass produced. In addition, on the 1st, 7th, 21st, and 35th day of the experiment, 48 eggs (4 eggs per replication) were randomly collected at 17:00 hours from each treatment and used to determine the egg quality at 20:00 hours. A dial pipe gauge (Ozaki MFG. Co., Ltd., Tokyo, Japan) was used to measure the eggshell thickness, excluding the inner membrane, which was determined to be based on the average thickness of the rounded end, pointed end, and the middle of the egg. The Haugh unit and yolk color were measured using an egg multi-tester (Touhoku Rhythm Co., Ltd., Tokyo, Japan). Eggshell color was determined using a color fan on a 1 to 15 scale (1 = light to 15 = dark brown) by a single trained evaluator. Eggshell strength was evaluated by an Eggshell force gauge, model II (Robotmation Co., Ltd., Tokyo, Japan).

Thirty-six birds were selected at random from each treat ment (3 hen per replication) on the 1st, 7th, 21st, and 35th day of the experiment. Blood samples were collected from the wing vein using a sterilized syringe with needle. Then half of the sample was transferred into either K_3_-ethylenediaminetetraacetic acid (EDTA) vacuum tubes or clot activator vacuum tubes (Becton Dickinson Vacutainer Systems, Franklin Lakes, NJ, USA) and stored at −4°C. Whole blood samples from the K_3_EDTA vacuum tube were analyzed for white blood cell (WBC), red blood cell (RBC), and lymphocyte concentrations using an automatic blood analyzer (ADVIA 120, Bayer, Tarrytown, NY, USA). For serum analysis, the clotted blood samples were centrifuged at 3,000×g at 4°C for 15 minutes to separate the serum. Total protein concentration in the serum was then analyzed using an automatic biochemistry blood analyzer (HITACHI747, Tokyo, Japan). The concentration of IGF-1 was analyzed using a Microplate reader (Versmax, Molecular device, USA).

On days 33 to 35, approximately 300 g excreta samples were collected from each replicate cage for quantifying ammonia (NH_3_), hydrogen sulfide (H_2_S), and total mercaptan (R-SH) according to the method provided by Dang et al [8]. The excreta samples were kept in 2-L sealed plastic containers for 3 days at room temperature (25°C). Each box had a small hole in the middle of one sidewall that was sealed by adhesive plaster. After the fermentation period, a Gastec (model GV-100) gas sampling pump was used for gas detection (Gastec Corp., Tokyo, Japan). The NH_3_, H_2_S, and R-SH concentrations were measured within the scope of 5.0–100.0 ppm (No. 3La, detector tube; Gastec Corp., Japan), 2.0 to 20.0 ppm (No. 4LK, detector tube; Gastec Corp., Japan), and 0.5 to 120.0 ppm (No.70 and 70-L, detector tubes; Gastec Corp., Japan), respectively. Before doing the measurement, the excreta samples were shaken manually for 30 seconds for homogenizing. The adhesive plaster was punctured, and 100 mL of head-space air was sampled at approximately 3 cm above the excreta surface.

### Statistical analyses

All data were subjected to statistical analysis in a randomized complete block design using the General Linear Model procedure of the SAS (Version 9.2., SAS Institute Inc., Cary, NC, USA), with each replicate cage being defined as the experiment unit. Orthogonal contrasts were used to examine the linear and quadratic effects in response to increasing the dietary supplementation of herbal mixture extract. The results were presented as means and pooled standard error of the mean (SEM). Probability values less than 0.05 were considered significant.

## RESULTS

A linear increase in egg weight in weeks 1 to 5 (p<0.05) and egg mass in week 1 (p<0.05) and weeks 1 to 5 (p<0.05), as well as a linear decrease in FCR in weeks 1 to 5 (p<0.05) were observed with the increase in the dose of YGF251 in the diet. However, dietary supplementation of YGF251 did not affect egg production rate and average daily feed intake (Table 2).

Eggshell strength on day 7 (p <0.05), 21 (p<0.01), and 35 (p<0.01), eggshell thickness on day 21 (p<0.05) and 35 (p< 0.01), and haugh unit on day 21 (p<0.01) and 35 (p<0.05) increased linearly with increasing YGF251 dose in the diet. However, the yolk color and eggshell color were not affected by YGF251 supplementation (Table 3).

Laying hens fed the diet supplemented with YGF251 increased serum IGF-1 concentration on day 21 (p<0.05; linearly) and 35 (p<0.01; linearly) and serum total protein concentration on day 35 (p<0.05; linearly) but did not affect the concentration of RBC, WBC, and lymphocytes (Table 4).

Supplementing YGF251 to the diet of laying hens decreased excreta ammonia emission (p<0.01; linearly) but did not affect the total mercaptan and hydrogen sulfide emission (Table 5).

## DISCUSSION

Feeding laying hens with a diet containing herbal active ingredients had positive effects on the production performance [14]. Yang et al [15] noted that the production performance improved in laying hens fed the diet supplemented with herbal active ingredients was due to the enhancement of serum IGF-1 concentration. A high circulating IGF-1 concentration in laying hens is related to a high egg weight [16], egg production rate [17], and feed efficiency [18]. Liu et al [19] reported that the increase of serum IGF-1 concentration in geese corresponded to the improvement of egg weight and egg production rate and the reduction of FCR. Sabry et al [20] observed that laying hens with higher serum IGF-1 concentration had a high egg production rate and egg mass and a low FCR. Similarly, in this study, laying hens fed the diet supplemented with YGF251 increased the egg weight, egg mass, and serum IGF-1 concentration, whereas decreased the FCR, in a dose-dependent manner. A low FCR means that laying hens absorb more nutrient ingredients from feed, which was beneficial to the improvement of production performance [21]. The serum total protein concentration reflects the utilization of protein from the feed [22]. In this study, dietary supplementation of YGF251 increased serum total protein level. Therefore, laying hens fed the YGF251 containing diet had good utilization ability of feed protein, which is manifested as the increase of serum total protein levels, which leads to low FCR, and further improving egg weight and egg mass. According to Kang et al [23], the serum IGF-1 level is positively associated with egg production rate. IGF-1 is an important regulator of vertebrate reproductive development [24]. As reported, the receptors of IGF-1 were distributed in the theca and granulosa cells [25]. IGF-1 can stimulate granulosa cells to secrete hormones [26], thus improving follicle maturation [27,28]. However, the diet supplemented with YGF251 did not affect the egg production rate of laying hens. According to reports, the effects of herbal active ingredients that promote IGF-1 secretion on egg production rate could vary with the egg production stage [29,30]. Han et al [31] reported that supplementing herbal active ingredients promoting IGF-1 secretion at the dose of 6 mg/kg resulted in a decrease in the egg production rate of quails aged 7 months, while it was found that the egg production rate of quails aged 12 months increased significantly. Therefore, that the egg production rate was not affected by YGF251 addition, may be due to the different effects of herbal active ingredients promoting IGF-1 secretion on egg production rate in different laying stages. Han et al [31] noted that the reason for this characteristic was that the IGF-1 regulates estrogen secretion differently in different laying stages. Despite YGF251 supplementation having no positive effects on the egg production rate of laying hens, there were no negative effects, which means there was no detraction from the potential of dietary supplementation of YGF251 to improve the production performance in laying hens. However, more studies are needed to evaluate the effects of YGF251 supplementation on production performance in different laying stages.

It is reported that IGF-1 can stimulate the secretion of ste roids by theca cells [32], thereby improving the synthesis of ovalbumin [33] and the quality of eggshell by promoting the development of the oviduct [34]. Therefore, the IGF-1 status of laying hens plays an important role in egg quality [24,35]. Mustafa et al [36] reported that the breed of quail with higher serum IGF-1 levels had higher haugh unit and eggshell thickness in egg quality than other breeds of quail. In this study, supplementing YGF251 to the diet of laying hens increased eggshell strength, eggshell thickness, and haugh unit but did not affect yolk color and eggshell color. The color of egg yolk is due to the accumulation of carotenoids in the egg. However, hens cannot synthesize carotenoids [37]. The results in the present study showed that dietary supplementation of YGF251 would not decrease consumer acceptance of eggs by damaging the egg yolk color. Besides, the eggshell is formed by calcium carbonate precipitation on eggshell membranes. Ca^2+^ and HCO_3_^−^ are not stored in the uterus before calcification of eggshell, rather they were continuously supplied via plasma through the transepithelial transport of the uterine endothelium during eggshell formation [37]. It is reported that IGF-1 produced higher rates of intestinal ion transport, thereby improving calcium absorption in animals [38]. However, the relationship between serum IGF-1 concentration and eggshell quality is unclear. It is speculated that the increase of serum IGF-1 concentration was beneficial to increase the transepithelial transport of calcium in the oviduct, thereby increasing the deposition of calcium on the eggshell membrane, thus improving eggshell strength and thickness. The haugh unit is a unit that expresses the degree of thinning of thick protein, which is related to the protein content in the thicker whites [39]. In an *in vitro* experiment, Duclos et al [40] and McFarland et al [41] reported that IGF-1 could promote the protein synthesis in chicken myotubes and turkey satellite cells. In addition, Kida et al [42] reported that IGF-1 has stimulating effect on ovalbumin synthesis in the oviduct cell culture of Japanese quails. Thus, the improvement of the haugh unit was related to the properties of IGF-1 promoting protein synthesis. In brief, laying hens fed the YGF251 containing diet could increase the acceptance of eggs by consumers through increasing haugh unit, eggshell thickness, and eggshell strength.

As we all know, the WBC, total protein, and lymphocytes in the blood play important roles in stimulating immune system of animals. High levels of WBC, total protein, and lymphocytes mean an improvement of immunity [21,43,44]. RBC are important cell types that transport oxygen. However, in our previous study [8], supplementation of YGF251 to the diet of broiler chicks did not affect the concentration of RBC, WBC, and lymphocytes. The present study finding shows that supplementing YGF251 in the diet of laying hens does not have any adverse effect on immune function.

The fecal gas emission came from the undigested nitrogen ingredients fermented by microflora in the intestine [45]. The improvement of nitrogen utilization rate reduces the substrate of microbial fermentation in the intestinal tract, thus reducing excreta noxious gas emission [46]. The serum total protein concentration is not only used as the indicator of animal immune efficiency, but also reflects the utilization of protein in the feed [21]. The reduction of serum total protein concentration indicates an inferior protein utilization in animals [47]. A high serum total protein concentration means a high feed protein utilization rate and low nitrogen excretion. This study observed that the serum total protein concentration increased in laying hens fed the diet supplemented with YGF251. Therefore, the decrease of excreta ammonia emission by supplementing YGF251 was related to the improvement of feed protein utilization, which showed an increase in serum total protein concentration.

## CONCLUSION

Supplementing YGF251 to the diet of laying hens improved the production performance (egg weight, egg mass, and FCR) and egg quality (haugh unit, eggshell thickness, and eggshell strength) by increasing serum IGF-1 concentration, as well as reduce the excreta noxious gas emission. The high egg weight, eggshell strength, eggshell thickness, and haugh units would increase the product value. The low excreta ammonia emission is beneficial to improve the barn environment. Therefore, YGF251 has potential as a production performance and egg quality enhancer and contribute to reducing excreta noxious gas emission.

## Figures and Tables

**Table 1:** Basal diet composition (as-fed basis)

Items	
Ingredients (%)	
Corn	50.4
Soybean meal	18.7
Wheat grain	10.0
Corn gluten meal	2.00
Wheat bran	5.00
Animal fat	4.40
Limestone	7.50
Dicalcium phosphate	1.40
Salt	0.30
Methionine	0.10
Vitamin premix^1)^	0.10
Mineral premix^2)^	0.10
Total	100.00
Analyzed nutrient composition	
Metabolizable energy (kcal/kg)	2,904
Crude protein (%)	15.00
Lysine (%)	1.80
Methionine (%)	0.32
Total calcium (%)	3.25
Total phosphorus (%)	0.61

1) Provided per kilogram of diet: 125,000 IU vitamin A; 2,500 IU vitamin D_3_; 10 mg vitamin E; 2 mg vitamin K_3_; 1 mg vitamin B_1_; 5 mg vitamin B_2_; 1 mg vitamin B_6_; 15 mg vitamin B_12_; 500 mg folic acid; 35,000 mg niacin; 10,000 mg Ca-Pantothenate and 50 mg biotin.

2) Provided per kilogram of diet: 8 mg Mn; 60 mg Zn; 25 mg Cu; 40 mg Fe; 0.3 mg Co; 1.5 mg I, and 0.15 mg Se.

**Table 2:** Effect of dietary supplementation of YGF251 on production performance in ISA Brown hens

Items	YGF251 (%)				SEM	p-value		
	
	0.00	0.05	0.10	0.15		Linear	Quadratic	Cubic
Egg production rate (%)								
Week 1	95.52	97.20	98.36	96.99	1.022	0.237	0.151	0.667
Week 2	97.39	97.15	97.01	97.02	0.758	0.721	0.869	0.983
Week 3	95.26	97.19	96.39	97.22	1.316	0.396	0.680	0.469
Week 4	96.18	97.19	97.19	97.82	0.785	0.176	0.812	0.645
Week 5	95.98	95.17	96.39	96.43	1.081	0.600	0.699	0.512
Weeks 1 to 5	96.06	96.78	97.07	97.10	0.705	0.295	0.632	0.958
Egg weight (g)								
Week 1	60.70	61.91	61.78	62.91	0.875	0.111	0.965	0.513
Week 2	60.79	61.86	61.39	61.95	0.480	0.175	0.600	0.242
Week 3	60.94	61.32	61.43	61.93	0.593	0.260	0.923	0.808
Week 4	60.98	61.26	61.25	61.09	0.457	0.878	0.644	0.942
Week 5	60.33	60.67	61.30	60.98	0.494	0.258	0.511	0.577
Weeks 1 to 5	60.75	61.40	61.43	61.77	0.275	0.020	0.577	0.449
ADFI (g/d/hen)								
Week 1	110.21	110.19	110.14	110.36	0.091	0.359	0.204	0.489
Week 2	110.17	110.07	110.10	110.41	0.149	0.281	0.189	0.805
Week 3	109.98	109.91	110.02	109.93	0.154	0.973	0.939	0.562
Week 4	110.02	109.69	109.67	109.74	0.240	0.422	0.410	0.844
Week 5	110.03	109.94	109.92	110.22	0.137	0.375	0.171	0.655
Weeks 1 to 5	110.08	109.96	109.97	110.13	0.082	0.678	0.099	0.952
FCR^1)^ (g feed/g egg)								
Week 1	1.91	1.83	1.81	1.81	0.034	0.051	0.297	0.785
Week 2	1.86	1.83	1.85	1.84	0.020	0.520	0.671	0.393
Week 3	1.90	1.85	1.86	1.83	0.035	0.212	0.798	0.493
Week 4	1.88	1.84	1.84	1.84	0.018	0.162	0.488	0.620
Week 5	1.90	1.91	1.86	1.88	0.022	0.212	0.855	0.268
Weeks 1 to 5	1.89	1.85	1.85	1.84	0.016	0.043	0.409	0.657
Egg mass (g/d/hen)								
Week 1	57.97	60.17	60.77	61.03	1.040	0.048	0.360	0.792
Week 2	59.21	60.10	59.53	60.11	0.629	0.457	0.807	0.366
Week 3	58.03	59.63	59.23	60.22	1.077	0.215	0.778	0.491
Week 4	58.65	59.52	59.53	59.75	0.551	0.196	0.562	0.667
Week 5	57.88	57.73	59.08	58.79	0.665	0.185	0.921	0.307
Weeks 1 to 5	58.35	59.42	59.63	59.98	0.519	0.040	0.497	0.667

SEM, standard error of the mean; ADFI, average daily feed intake; FCR, feed conversion ratio.

1) Feed conversion ratio was calculated as average egg weight to the average daily feed intake ratio.

**Table 3:** Effect of dietary supplementation of YGF251 on egg quality in ISA Brown hens

Items	YGF251 (%)				SEM	p-value		
	
	0.00	0.05	0.10	0.15		Linear	Quadratic	Cubic
Eggshell strength (kg/cm^2^)								
Day 1	3.21	3.21	3.34	3.05	0.165	0.619	0.376	0.441
Day 7	3.36	3.42	3.73	3.84	0.166	0.021	0.871	0.541
Day 21	2.83	3.53	3.78	3.96	0.173	<0.001	0.135	0.606
Day 35	2.98	3.54	3.81	4.17	0.175	<0.001	0.578	0.638
Eggshell thickness (10^−2^ mm)								
Day 1	37.80	37.20	37.00	37.35	0.501	0.491	0.346	0.947
Day 7	39.57	39.32	40.18	40.38	0.547	0.179	0.682	0.468
Day 21	38.70	39.83	40.23	40.52	0.494	0.010	0.392	0.781
Day 35	38.45	40.15	40.35	40.63	0.445	0.001	0.116	0.429
Yolk color								
Day 1	8.15	8.00	8.20	7.95	0.142	0.531	0.726	0.212
Day 7	8.75	8.85	8.75	8.60	0.099	0.217	0.210	0.735
Day 21	8.60	8.60	8.75	8.40	0.126	0.427	0.169	0.253
Day 35	8.65	8.55	8.40	8.60	0.123	0.588	0.228	0.470
Eggshell color								
Day 1	11.10	11.20	11.15	10.95	0.350	0.751	0.670	1.000
Day 7	11.95	12.25	12.20	12.40	0.309	0.349	0.872	0.665
Day 21	11.75	11.05	11.90	11.10	0.415	0.262	0.282	0.915
Day 35	11.45	11.20	11.35	11.00	0.228	0.242	0.827	0.379
Haugh unit								
Day 1	90.48	88.97	90.61	89.13	0.795	0.502	0.985	0.083
Day 7	91.74	91.93	91.98	92.77	0.777	0.366	0.700	0.803
Day 21	88.80	90.69	91.39	91.81	0.660	0.002	0.266	0.759
Day 35	87.87	88.81	90.39	92.01	1.278	0.017	0.786	0.919

SEM, standard error of the mean.

**Table 4:** Effect of dietary supplementation of YGF251 on blood hematology in ISA Brown hens

Items	YGF251 (%)				SEM	p-value		
	
	0.00	0.05	0.10	0.15		Linear	Quadratic	Cubic
IGF-1 (ng/mL)								
Day 1	35.00	35.66	35.56	34.80	1.185	0.897	0.558	0.985
Day 7	35.38	37.50	39.76	41.04	2.038	0.051	0.839	0.904
Day 21	35.06	37.20	38.58	40.08	1.702	0.046	0.853	0.909
Day 35	35.18	40.60	42.30	43.36	1.245	<0.001	0.100	0.588
Total protein (g/dL)								
Day 1	5.18	5.10	5.02	4.96	0.168	0.340	0.953	0.979
Day 7	4.96	5.14	5.16	5.16	0.107	0.212	0.411	0.773
Day 21	4.72	5.00	5.06	5.16	0.207	0.155	0.669	0.782
Day 35	5.44	5.74	5.84	6.18	0.242	0.047	0.935	0.689
RBC (10^6^/μL)								
Day 1	2.09	2.07	1.92	2.08	0.087	0.663	0.325	0.277
Day 7	2.01	1.91	2.00	1.86	0.064	0.243	0.713	0.166
Day 21	1.85	1.87	1.91	1.89	0.072	0.634	0.805	0.741
Day 35	1.78	1.70	1.71	1.83	0.073	0.631	0.174	0.952
WBC (10^3^/μL)								
Day 1	377.42	351.90	296.92	363.48	30.985	0.495	0.157	0.292
Day 7	317.64	282.74	301.02	281.16	22.128	0.371	0.738	0.370
Day 21	266.26	259.96	271.04	259.58	23.279	0.933	0.913	0.706
Day 35	214.18	214.44	208.98	213.57	21.737	0.941	0.922	0.873
Lymphocyte (%)								
Day 1	75.80	74.60	82.00	76.40	3.545	0.570	0.544	0.192
Day 7	73.40	77.80	78.40	77.60	4.339	0.506	0.558	0.903
Day 21	73.20	75.40	75.60	68.00	4.230	0.428	0.264	0.763
Day 35	76.20	79.60	71.40	77.20	3.388	0.736	0.728	0.110

SEM, standard error of the mean; IGF-1, insulin-like growth factor-1; RBC, red blood cell; WBC, white blood cell.

**Table 5:** Effect of dietary supplementation of YGF251 on fecal gas emission in ISA Brown hens

Items (ppb)	YGF251 (%)				SEM	p-value		
	
	0.00	0.05	0.10	0.15		Linear	Quadratic	Cubic
NH_3_	26,116.99	19,857.55	17,051.59	12,202.32	2.940	0.008	0.764	0.731
R-SH	4,093.13	3,660.94	3,447.39	2,583.00	0.822	0.249	0.811	0.830
H_2_S	4,737.40	2,368.70	2,153.36	2,799.37	0.768	0.123	0.087	0.731

SEM, standard error of the mean; NH_3_, ammonia; R-SH, total mercaptan; H_2_S, hydrogen sulfide.
